# A new species of *Metaeuchromius* (Lepidoptera, Crambidae) from the Tibetan glacier area of China

**DOI:** 10.3897/zookeys.475.8766

**Published:** 2015-01-22

**Authors:** Wei-Chun Li, Dong Liu

**Affiliations:** 1 College of Agronomy, Jiangxi Agricultural University, Nanchang, 330045, China

**Keywords:** Pyraloidea, Crambinae, taxonomy, Qinghai-Tibetan Plateau

## Abstract

*Metaeuchromius glacialis* Li, **sp. n.** is described from the Tibetan glacier area of China. The new species is similar to *Metaeuchromius circe* Bleszynski by the distal projection of costa exceeding the apex of valva, and the phallus with strong spine-like cornuti in the male genitalia. Images of male adult, tympanal and scent organs as well as genitalia of the new species are provided.

## Introduction

*Metaeuchromius* was established with *Eromene yuennanensis* Caradja, 1937 as the type species (Bleszynski 1960). The genus has fourteen species with a Palearctic and Oriental distribution (Bleszynski 1965; Park 1990; Schouten 1997; Nuss and Speidel 1999; Chen et al. 2002; Sasaki 2005; Li et al. 2009; Nuss et al. 2014). Prior to this study, eight species of *Metaeuchromius* were recorded in China (Li et al. 2009). However, the known species are rarely recorded around the monsoon temperate glaciers of the south-eastern part of the Qinghai-Tibetan Plateau in China. Moreover, it is becoming more and more critical to pay close attention to the species living around mountain glaciers because global warming is highly likely to cause changes in the biodiversity of these regions (Li and Liu 2014). In the present paper, *Metaeuchromius glacialis*, collected from Galongla Mountain, Tibetan glacier area, is described as new to science.

## Material and methods

Specimens were collected by a 250-W high-pressure mercury lamp. They were hand-collected alive and killed by ammonium hydroxide just prior to mounting and spreading (Landry and Landry 1994). The type specimens of *Metaeuchromius* species are deposited in the Natural History Museum of London (NHM) have been examined by corresponding author. All the type specimens of the new species are deposited in the Insect Museum, Jiangxi Agricultural University, Nanchang, China (JXAUM).

The terminology for the terminal dots formula of the forewings follows Schouten (1997), the terminology for the tympanal organs follows Maes (1985) and Nuss (2005).

Genitalia preparation followed the standard procedure, using boiling 10% KOH solutions to digest internal tissues; after careful cleaning and removal of scales, genitalia were examined, compared, and described before being mounted on microscope slides by corresponding author. The images of the adults were taken with a digital camera (Canon G12). The illustrations of the genitalia were prepared with a digital camera DV320 OPTPro2010_Chs attached to a digital microscope Optec BK-DM320.

## Taxonomic account

### Metaeuchromius glacialis

Taxon classificationAnimaliaLepidopteraCrambidae

Li sp. n.

http://zoobank.org/D38737BC-E4CF-432C-A1D4-ECFB14E07C8E

Figs 1–3

#### Holotype.

♂, China, Tibet, Medog, the foot of Mt. Galongla (29°44.2947'N, 95°40.6068'E), 3415 m, 22.VII.2014, coll. Wei-Chun Li and Dong Liu et al.

**Paratypes.** 4 ♂♂, same data as the holotype except dated 20–23.VII.2014.

#### Diagnosis.

**Figures 1–3.**
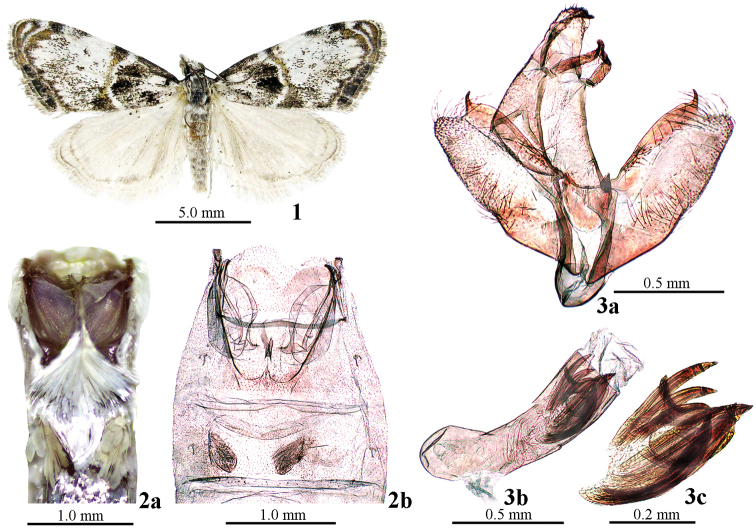
*Metaeuchromius glacialis* Li, sp. n. **1** Adult, Holotype **2** Abdomen, paratype **a** Tympanal and scent organs with scales **b** Tympanal and scent organs without scales **3** Male genitalia, paratype **a** Without phallus **b** Phallus **c** Cornuti.

**Figures 4–6.**
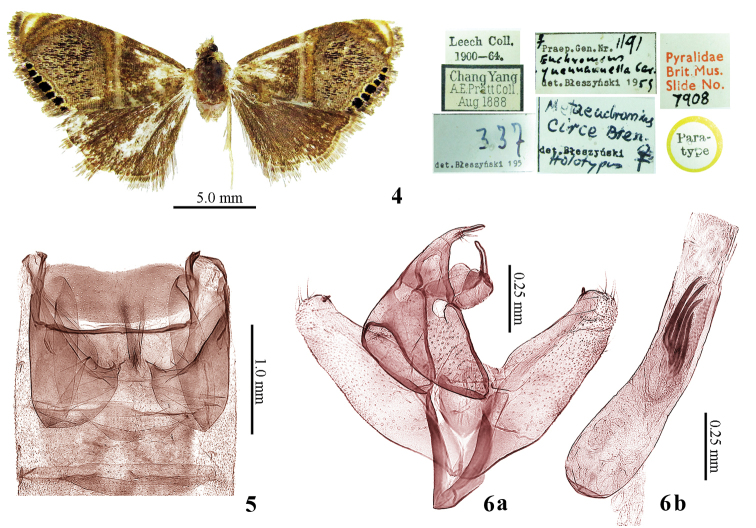
*Metaeuchromius circe* Bleszynski**. 4** Adult, paratype, female **5** Tympanal organ **6** Male genitalia, additional specimen **a** Without phallus **b** Phallus.

In male genitalia, this new species is similar to *Metaeuchromius circe* Bleszynski, 1965 in the distal projection of costa exceeding the apex of valva, and the phallus with strong spine-like cornuti. Based on a comparison with the type specimens and additional specimens (2 ♂♂, China, Hunan Province, Shimen County, Mt. Huping, ca. 1200 m, 18.VII.2013, coll. Wei-Chun Li et al.) of *Metaeuchromius circe*, the new species can be distinguished by the forewing with a conspicuously convex medial fascia and terminal dots with formula 2-3-1 (Fig. 1) whereas the medial fascia of *Metaeuchromius circe* is straight and the terminal dots with formula 2-3-2 (Fig. 4). The new species has the saccus tympani of male tympanal organ extending to two thirds of the second sternite and the third sternite with two scent organs (Fig. 2) while the saccus tympani of *Metaeuchromius circe* exceeds the posterior margin of the second sternite and there is no scent organ (Fig. 5). The male genitalia of the new species have a strong spine-like projection at the end of costa of valva and the phallus with eight cornuti (Fig. 3) while *Metaeuchromius circe* only has a small pointed costal tip and four cornuti (Fig. 6).

#### Description.

Adult (Fig. 1). Forewing length 9.0–10.0 mm. Frons pale brown. Vertex white except pale brown in middle. Labial palpus approximately one and half as long as compound eye’s diameter, pale brown. Maxillary palpus pale brown, distally white. Antenna scapus white; flagellomere pale brown. Patagium and thorax white. Tegula blackish brown mixed with white, posterior margin with long and thin white scales. Forewing densely covered with blackish brown scales from basal one fifth to medial fascia, the other area sparsely suffused with pale brown scales; costa with longitudinal blackish brown stripe extending from base to near medial fascia; medial fascia conspicuously convex, incurved slightly near middle, running to before middle of dorsum, golden, edged with pale brown; discoidal cell with two brown spots; apex golden mixed with pale brown, with one white stripe; subterminal line golden mixed with pale brown; six terminal black dots running from middle of termen to tornus, fomula 2-3-1, each group delimited by white, each dot of a group divided by golden; cilia pale brown. Hindwing white, suffused with gray scales; subterminal fascia pale brown, inconspicuous; cilia white. Fore- and midlegs pale brown, tarsi with white rings; hindleg yellowish white.

**Male abdomen** (Fig. 2). Praecinctorium with a cluster of slender white scales; bulla tympani of tympanal organ open, bean-shaped, inner margin convex anteriorly and concave posteriorly; saccus tympani broad, rounded, extending to two thirds of second sternite; venula secunda present. Third sternite with two clusters of yellowish white scales on lateral side (Fig. 2a), bearing two oblong scent organs, opening towards lateral side, outside wall with pits, a cluster of slender scales attached to pits on inward tip (Fig. 2b).

**Male genitalia** (Fig. 3). Uncus curved downward, tapering to blunt apex. Gnathos curved upward slightly, distally rounded. Tegumen arms approximately twice as long as gnathos. Valva broad basally, narrowed towards apex; apex rounded. Costa strongly sclerotized, ending with a strong spine-like projection directed upward, reaching apex of valva. Juxta basally convex, gently broadened towards tip; distal margin incurve and forming two triangular lateral projections. Phallus nearly as long as valva; cornuti composed of eight strong spines.

Female unknown.

#### Distribution.

This species is only known from Galongla Mountain, in Medog County, Tibet, China.

#### Natural history.

**Figure 7.**
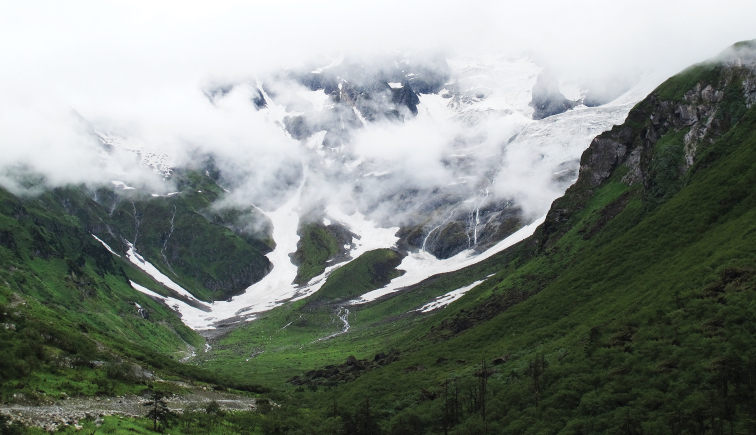
Natural environment of collecting localitiy of *Metaeuchromius glacialis* Li, sp. n.

Unknown except that the moths fly late July and are attracted to light. The habitat in which this species has been collected is located at an altitude of 3415 m, at the foot of Galongla Mountain. Most parts of the mountain are covered with snow; the vegetation on the south slope is a blend of alpine meadows, shrubs and conifer trees (Fig. 7).

#### Etymology.

The specific name is derived from the Latin *glacialis* = glacier, in reference to the species occurance in the Tibetan glacier area.

## Supplementary Material

XML Treatment for Metaeuchromius glacialis
